# Reaching those at risk: Active case detection of leprosy and contact tracing at Kokosa, a hot spot district in Ethiopia

**DOI:** 10.1371/journal.pone.0264100

**Published:** 2023-06-21

**Authors:** Tsehaynesh Lema, Kidist Bobosha, Christa Kasang, Azeb Tarekegne, Saba Lambert, Addis Mengiste, Sven Britton, Abraham Aseffa, Yimtubezenash Woldeamanuel

**Affiliations:** 1 College of Health Science, Addis Ababa University, Addis Ababa, Ethiopia; 2 Armauer Hansen Research Institute (AHRI), Addis Ababa, Ethiopia; 3 All African Leprosy, Tuberculosis, Rehabilitation and Training (ALERT) Center, Addis Ababa, Ethiopia; 4 German Leprosy and TB Relief Association (GLRA), Würzburg, Germany; 5 London School of Hygiene and Tropical Medicine, London, United Kingdom; 6 Department of Medicine, University Karolinska Institutet, Stockholm, Sweden; Tanta University Faculty of Medicine, EGYPT

## Abstract

**Introduction:**

Leprosy is a chronic mycobacterial disease of public health importance. It is one of the leading causes of permanent physical disability. The prevalence of leprosy in Ethiopia has remained stagnant over the last decades. The aim of the study was to identify new leprosy cases and trace household contacts at risk of developing leprosy by active case detection. The study area was Kokosa district, West Arsi zone, Oromia region, Ethiopia.

**Method:**

A prospective longitudinal study was conducted from June 2016-September 2018 at Kokosa district. Ethical approvals were obtained from all relevant institutions. Health extension workers screened households by house-to-house visits. Blood samples were collected and the level of anti-PGL-I IgM measured at two-time points.

**Results:**

More than 183,000 people living in Kokosa district were screened. Dermatologists and clinical nurses with special training on leprosy confirmed the new cases, and their household contacts were included in the study. Of the 91 new cases diagnosed and started treatment, 71 were recruited into our study. Sixty-two percent were males and 80.3% were multibacillary cases. A family history of leprosy was found in 29.6% of the patients with cohabitation ranging from 10 to 30 years. Eight new leprosy cases were diagnosed among the 308 household contacts and put on multi-drug therapy. The New Case Detection Rate increased from 28.3/100,000 to 48.3/100,000 between 2015/2016 and 2016/2017. Seventy one percent of leprosy patients and 81% of the household contacts’ level of anti-PGL-I IgM decreased after treatment. In conclusion,the results of the study showed the importance of active case detection and household contact tracing. It enhances early case finding, and promotes early treatment, thereby interrupting transmission and preventing potential disability from leprosy.

## Introduction

At the end of 2021, the World Health Organization (WHO) reported globally 127,396 new cases of leprosy. Of these 7,198 had grade-two disability (G2D) and 8,629 were new child leprosy cases. Ethiopia reports greater than 1000 new cases of leprosy yearly. The registered new cases in 2020 numbered 2,591. Ethiopia ranks 7^th^ among the 23 global priority countries and 3^rd^ in Africa with 390 new child cases and 384 G2D cases per year [1].

Early diagnosis and treatment are the cornerstones of leprosy management, preventing permanent disabilities and reducing transmission. Continuous long-term follow-up contributes to early diagnosis. WHO member countries adopted the global 2016–2020 strategy which moves towards a leprosy-free world [2], This was recently updated in the “Towards zero leprosy (2021–2030)” strategy [3], which includes active case detection (ACD) as one of its pillars. The scale-up of leprosy control through ACD is achievable, leading to interruption of transmission and potential elimination of disease [3].

The risk of developing leprosy increases among persons with prolonged contact, with diagnosed cases, but also in particular with new untreated leprosy patients. In addition, risk increases when contacts live with the patients [4]. Studies show the contacts of leprosy cases are 6 fold more likely to develop disease than those in the general population. Contacts of multibacillary (MB) patients have 8-fold increased risk compared to the paucibacillary (PB) contacts [5]. Hence, screening household contacts (HHCs) is crucial for possible early case detection.

Serum anti-PGL-I antibody levels in HHCs of leprosy patients contribute to ACD. Studies from Brazil demonstrated the usefulness of serum anti PGL-I antibody levels in HHCs. Spencer *et al*. showed that many HHCs developed increased titers over time and this was associated with development of borderline lepromatous leprosy (BL) after two years routine follow-up [6]. However, anti-PGL-I antibody levels are higher in MB than PB leprosy patients, which may limiting the usefulness of anti-PGL-I as a prognostic biomarker to MB patients.

In 2003, Ethiopian Federal Ministry of Health (FMoH) initiated a new program, “Accelerated Expansion of Primary Health Care”, which promoted the use of Health Extension Workers (HEW)s to provide basic package health services to the rural community.

In this study, the engagement of HEWs was vital to conduct the house-to-house screening and contact tracing. The HEWs assigned to work in the community were trained to identify patients with the major signs and symptoms of leprosy. Leprosy suspects were refered to health centers for further screening, confirmation and management.

This study designed to demonstrate the implementaton of ACD in identifing new leprosy patients and fill the gap observed by passive case detection. Additionally, we aimed to determine whether early management of leprosy patients will reduce the risk of developing leprosy complications. This study was conducted at one of the leprosy hotspot areas of the country.

## Materials and methods

A prospective longitudinal study was conducted in Kokosa district. The study period was from June 2016 to September 2018. The district is located in the West Arsi Zone, Oromia region. Oromia is the largest region of Ethiopia. It accounts for 36.7% of the population but comprises 57% of new cases of leprosy in the country [7]. When the study began the district had a population of 183,685 with 36,495 HHCs (Source: West Arsi Zone Health Bureau). There were 8 health facilities. They include Hogiso, Hebano, G/Hurufa, Boro, Bokore, Kokosa, Ararso, and Kokosa hospital. The hospital participated in the diagnosis of some of the leprosy cases who were referred back to the nearest study health centers for treatment, proper management, and follow-up.

The source population was all individuals with dermatological complaints from Kokosa district. Leprosy suspects were screened and referred to health centers for further examinations and confirmation. Consenting HHCs of the confirmed leprosy patients were included in the follow-up study. A pre-structured questionnaire was completed and blood samples from patients and HHCs collected. Slit skin smear (SSS) was collected from patients only. Tuberculosis (TB) and leprosy screened endemic controls (ECs) were recruited from the same population.

### Definition of terms

**New leprosy patient**: a patient with MB or PB leprosy who has never had previous treatment for leprosy [8].

**Treatment completed:** a patient who has completed the full regimen of MDT within the prescribed period; the full regimen is 12 months of therapy within a 15 month period for MB cases and 6 months of therapy within a 9 month period for PB patients [8].

**Household contacts (HHCs):** individuals who have been living at least 6 months or more with an index case, and without signs and symptoms of leprosy at enrolment.

**Endemic controls (ECs):** apparently healthy individuals who have lived in the district for 2 years. ECs have had no contact with an index case and no previous treatment history of leprosy or TB.

### Biological samples preparation

At the first-time point, a total of 149 blood samples were collected from 24 leprosy patients, 100 HHCs, and 25 ECs. 103 blood samples were collected from 88 HHCs and 15 leprosy patients during the second visit. Nine patients and 12 HHCs were lost to follow-up. Clinical samples that included Slit skin smear (SSS) and biopsy samples from confirmed leprosy patients were collected. Also, blood samples from leprosy patients, HHCs, and ECs were collected.

Slit skin smear (SSS) reading was done at the Armauer Hansen Research Institute (AHRI). Three SSS from lesions of each leprosy patients were taken. The smears were stained with Ziehl-Nelson. The average number of bacilli per microscopic field was termed bacterial index (BI) and used for reporting. An oil-immersion objective was used for microscropy.

The Up-converting Phosphor Lateral Flow Assay (UCP-LFA) was used to analyze anti-PGL-I IgM antibodies for both time points, and was performed by Leiden University Medical Center (LUMC).

Anti-PGL-I IgM was measured before and after treatment for leprosy patients. It was also done for HHCs before and after their paired index cases completed treatment. The measurement helps to identify the biomarker signature specific for leprosy disease. IgM antibodies against *M*. *leprae* PGL-I were detected using conjugate antigen comprised of the natural disaccharide of PGL-I linked to human serum albumin (NDO-HSA 500 ng/ well in 50 μl). The antigens provided through the NIH/NIAID Leprosy (Contract N01-AI-25469) as described earlier [9]. Serum dilutions (50 μl/ well; 1:800) were incubated at room temperature (RT).The incubation was for 120 minutes in flat-bottomed microtiter plates (Nunc) coated with NDO-HSA. After washing, diluted enzyme-linked secondary antibody solution used. The solution (anti-human IgG/IgM/IgA–HRP; Dako, Heverlee, Belgium; 50 μl/ well) was added to all wells for 120 minutes at room temperature. After washing, diluted TMB solution (50 μl/ well) was added to all wells and plates were incubated in the dark for 15 minutes at RT. The reaction was stopped by adding 50 μl/well 0.5 N H_2_SO_4_. Absorbance was determined at a wavelength of 450 nm. Samples with a net optical density at 450 nm (OD) above 0.149 were considered positive.

Positive and negative control serum samples was used to check ELISA performance. The diagnostic potential of anti-PGL-I IgM was evaluated based on the clinical classifications. UCP-LFA results of anti-PGL-I IgM assay is displayed as the Ratio value (R) of patient to control sample. After one year, the Ratio of anti-PGL-I IgM in HHCs was determined for the first and second time screening. High responses were defined as those with optical density (OD) value above 1.195.

Case record forms were used to collect personal and clinical data, and all data was double entered into an MSAccess database. Epi-info software was used for verification and cleaning. STATA/SE version 15 (College Station, TX, USA) used for statistical analysis.

The study proposal evaluated and approved by multiple ethical review boards, including AHRI/ALERT Ethical Review Committee (AAERC PO37/2014), the National Health Research Ethics Review Committee (NHRERC 3-10/014/2015), the College of Health Sciences Institutional Review Board, Addis Ababa University (AAU,002/15 DMIP), and the Oromia Regional Health Bureau Ethical Review Committee (BEFO/HBTFH/1-8/2416).

Study participants were informed about the study objectives, and each participant signed informed consents. Parents of children aged 12–17 signed consent forms if their children were willing.

## Results

Dermatologists and leprosy experts’ clinical assessment were used as the main diagnostic criteria. FMoH guidelines defined the diagnosis, based on the three cardinal signs of leprosy: hypopigmented or reddish lesions with loss of sensation; enlarged peripheral nerves and acid fast bacilli (AFB) identified in SSS.

**Fig 1:**
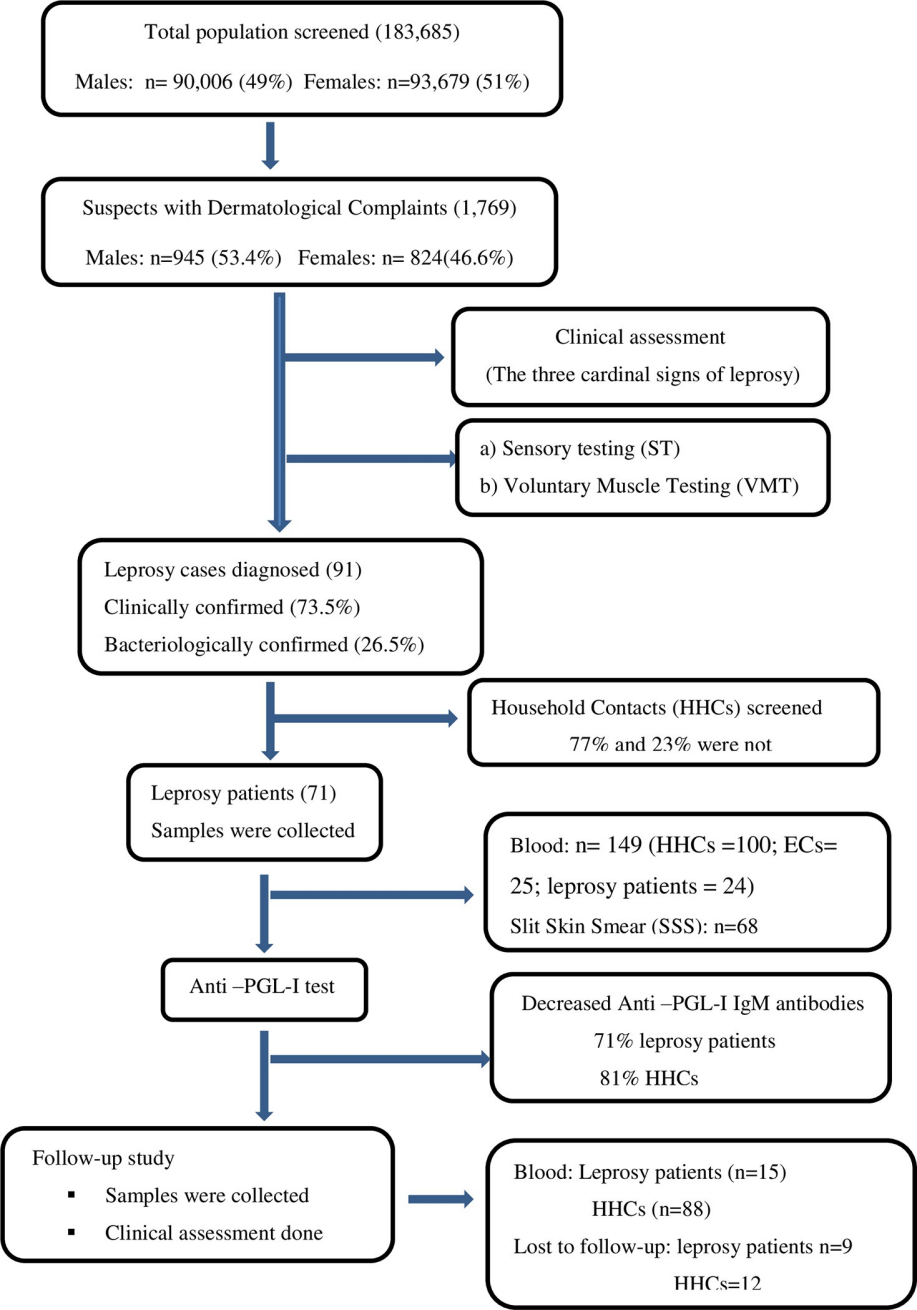
Flow diagram of study participant’s recruitment at Kokosa District.

During the study period, 91 new leprosy cases were confirmed out of 1,769 suspects. Seventy-one patients were detected through active household level screening and contact tracing. The other 20 patients’ self-presented at health facilities seeking treatment. After proper orientation 71 were enrolled in the study. Blood samples were collected before starting leprosy treatment (Fig 1).

Of the 71 new cases, 308 HHCs (77%) were screened. Of these, eight new leprosy cases from six index leprosy cases were diagnosed and initiated on MDT. Most of the HHCs (51.4%) were below the age of 15. 26.2% of the HHCs were from Hebeno kebele and the male to female proportion was almost equal.

**Fig 2:**
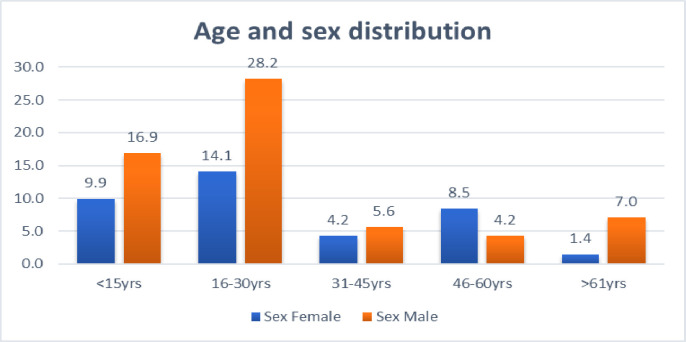
Age and sex distribution of patients affected by leprosy in Kokosa District. X-axis represents age group and Y-axis represents frequency.

The age and sex distribution of patients affected by leprosy is depicted in Fig 2. Of the new diagnosed cases starting treatment, 62% (44/71) were males and 38% (27/71) were females (Fig 2). The age ranged from 4 to 70 years and 42.3% (30/71) of the patients were aged between 16 and 30 years Sociodemographic and PGL-I Raw Data 1 in S1 File.

Nearly all patients, 95.8% (68/71), resided in remote rural areas; three lived in rural towns. 59.2% (42/71) were illiterate. Most of the leprosy patients, 88.7% (63/71), were diagnosed at the health centers. 11.2% (8/71) were diagnosed at the Kokosa hospital. Among the 71 index cases, 59.1% of them had a household size from six to ten consistent with crowding (Table 1).

**Table 1:** Socio-demographic characteristics of patients affected by leprosy at enrollment, Kokosa district, 2016–2017.

Variables		Frequency n = 71	Percent (%)
**Age in years**	<15	19	26.8
	**16–30**	**30**	**42.3**
	31–45	7	9.9
	46–60	9	12.7
	>61	6	8.5
**Sex**	**Male**	**44**	**62**
	Female	27	38
**WHO classification**	**Multibacillary (MB)**	**57**	**80.3**
	Paucibacillary (PB)	14	19.7
**Residence**	Urban	3	4.2
	Rural	68	95.8
**Education**	**Not able to read & write**	**42**	**59.2**
	Able to read and write	5	7.04
	Primary	16	22.5
	Secondary	7	9.9
	College	1	1.4
**Household size number**	<5	23	32.4
	**6–10**	**42**	**59.1**
	11–15	0	0
	16–20	5	7.1
	>21	1	1.4

### Clinical features of persons affected by leprosy

**Fig 3:**
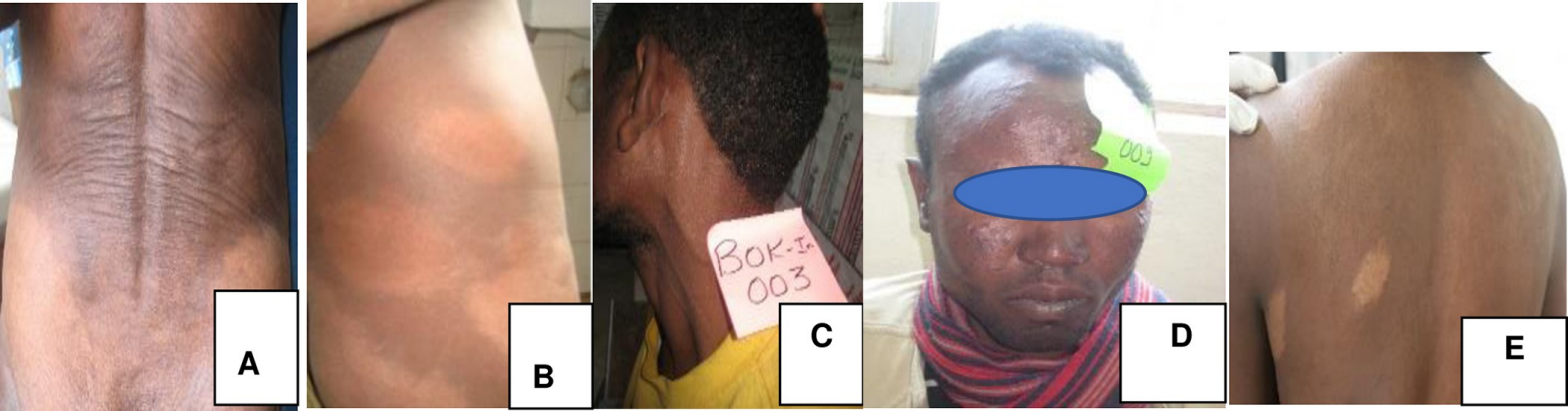
Leprosy patients enrolled in Kokosa ACD study. A and B = MB patients.>5 cutaneous lesions; C = PNL, no cutaneous lesions; D = MB (LL) with numerous nodules; E = PB with< 5 cutaneous lesions [(Photos by Tsehaynesh Lema (PI)].

Among the 71 enrolled patients, 80.3% (57/71) were MB while the remaining 19.7% (14/71) were PB according to WHO classification (Fig 3).

**Fig 4:**
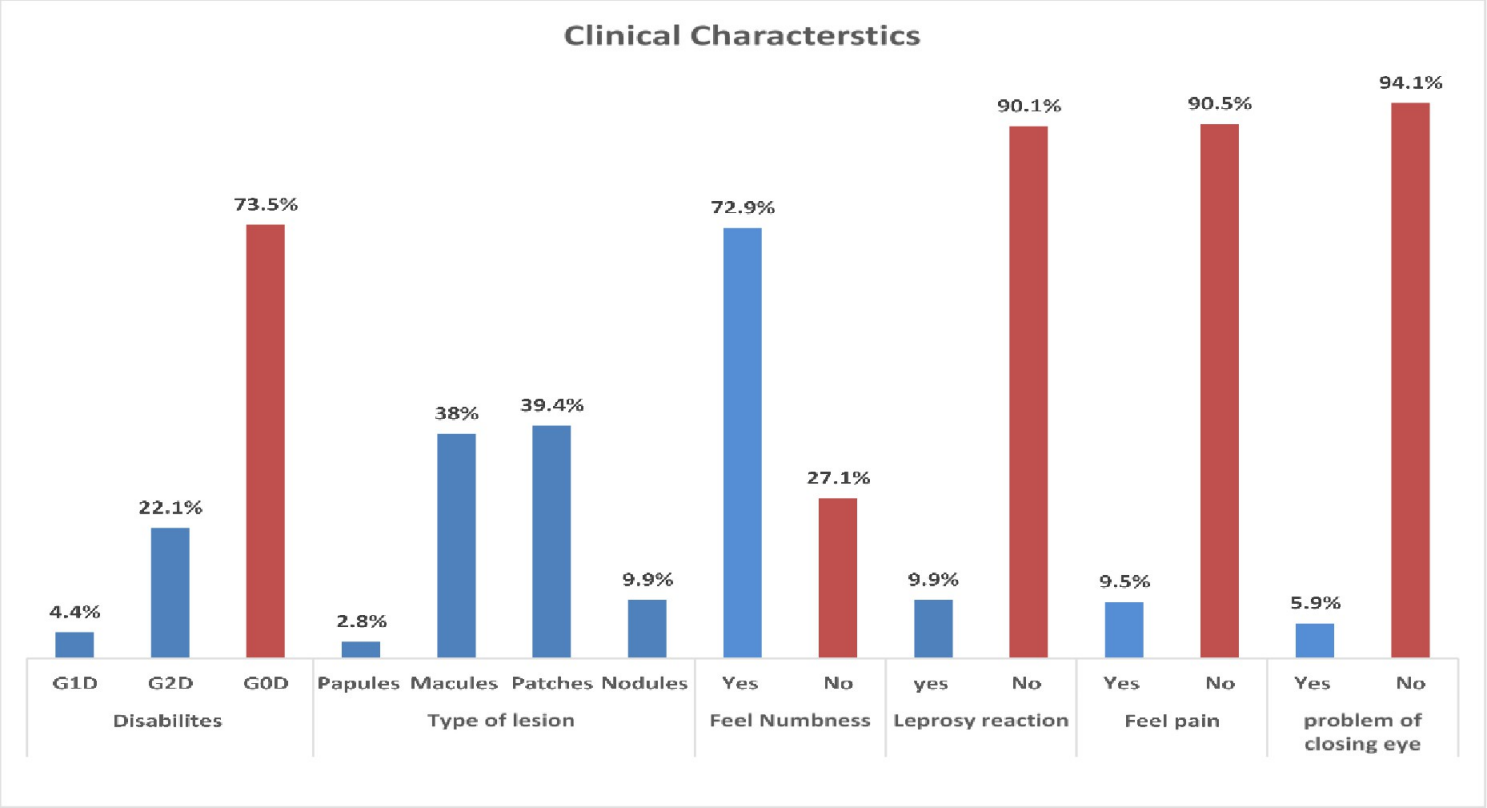
Clinical characteristics of patients at enrolment. G0D = grade 0 disabilities, G1D: Grade 1 disabilities, G2D: Grade 2 disabilities, ***9.9% of the patients are PNL cases without cutaneous manifestations.

Most of the patients (72.9%) presented with numbness and 39.4% had patches. Very few (9.5%) reported having pain at the hypopigmented lesion. 5.9% had difficulties closing their eyes and 9.9% presented with leprosy reactions. Five of them had Type I reactions (T1R) and two had Erythema Nodosum Leprosum (ENL) reactions (Fig 4).

#### Disabilities observed at diagnosis

**Fig 5:**
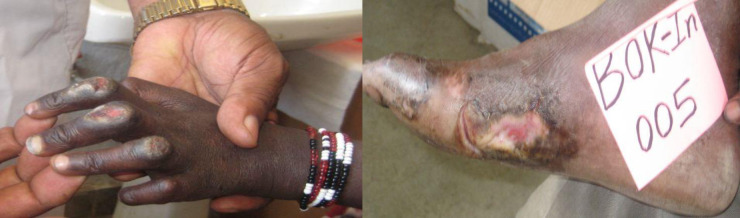
Leprosy patients diagnosed with G2D in Kokosa ACD study [(Photos by Tsehaynesh Lema (PI)].

Disability is one of the complications of leprosy that includes impairment, activity limitation, or participation restriction affecting a person (Fig 5). Most disabilities and deformities due to leprosy result directly or indirectly from loss of peripheral nerve function secondary to infection of the nerves supplying the eyes, hands, and/or feet. In this study, 4.2% (3/71) of the patients had grade 1 disabilities (G1D), including 2 MB and 1 PB patients. 21.1% (15/71) of the patients had grade 2 disabilites (G2D), 2 PB, 3 pure neural leprosy (PNL) and 10 MB patients.

#### Slit-skin smears

In this study, the positive SSS was 26.5% (18/68) with BI ranging from 1–6. Half of the smears were negative (34/68). Of which 3 were from PNL patients, 9 were from PB patients and 22 were from MB patients. SSS was not done for 23.5% (16/68) of the leprosy patients among which 5 PB, 4 PNL, and 7 were MB patients.

#### Anti-PGL-1 IgM antibody

**Fig 6:**
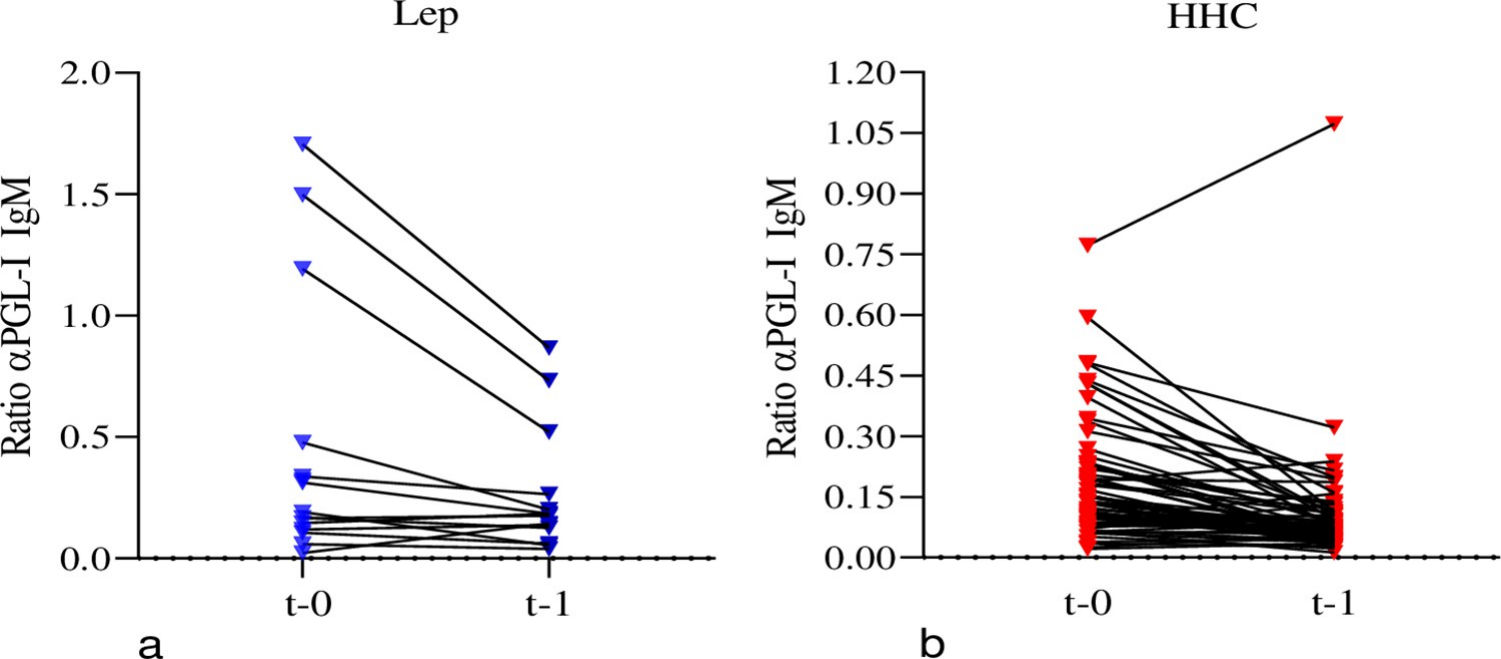
Level of biomarkers before and after treatment. a) The levels of anti-PGL-I IgM in the plasma of leprosy patients before and after MDT. b) The levels of anti-PGL-I IgM among HHCs at enrollment and after a year (after the index patients completed MDT, 12 months for MB and 6 months for PB).

A total of 149 whole blood samples were collected from the cross-sectional study and 103 from the longitudinal study. Both sample sets were analyzed by assays that included an anti-PGL-I-IgM antibody assay. The level of anti-PGL-I IgM decreased after treatment. 71% of the leprosy patients (Fig 6A) and 81% of the HHCs (Fig 6B and Sociodemographic and PGL-1 raw data 2 in S1 File) showed decreased levels. Most of the participants’ anti-PGL-I-IgM antibodies levels were below 0.5 O.D. In contrast, three patients had significant expression of anti-PGL-I-IgM antibody (O.D> 1.2) at baseline, and these levels descreased substahntially after MDT.While most HHCs showed lower levels of anti-PGL-I-IgM antibodies at baseline than index cases, one HHC showed elevated levels.

## Discussion

Active case detection (ACD) of leprosy is a key intervention, reaching affected patients early and hence prevents the diseae spread. We used a house-to-house screening approach for ACD which appeared to be highly effective. Whereas 52 new cases were reported in the year prior to the study initiation (2015/2016), 91 new cases were reported in the 12 months of this study (2016/2017). However, in the years following the survey, when active case detection was no longer being performed, the new case detection showed a declining trend. In 2017/2018, 2018/2019, 2019/2020 reports, 54, 21 and 24 new patients were registered, respectively. This dramatic spike in case detection in the study year strongly suggests the importance of ACD through the active involvement of HEWs. A very recent study in the Eastern Hararghe applied ACD and contact screening, also revealed the importance of active rather than passive case detection [10].

The current study indicated 29.6% of the diagnosed patients had at least one leprosy family contact, with HHCs remaining in contact with varying duration ranging from < 10 to 30 years. Previous studies have shown that HHCs have 4–9 times increased risk of acquiring the disease. In one study, two contact-related factors mentioned, including closeness and intensity of the contact to the index leprosy case [5].

In this study, eight of the 308 HHC screened were diagnosed with leprosy, a much greater prevalence that was expected from the population at large. This shows the importance of close exposure to the leprosy patient as an important risk factor. Our findings are in agreement with other studies showing the importance of both ACD and contact tracing [11–13] compared with passive case finding [11].

Among the 71 indexes, 59.1% of them had a houshold size from six to ten, indicating crowding (Table 1). The study of Kiribati *et al* have emphasized the importance of crowding, with at least 7 persons in a household considered as a risk for leprosy infection [14]. Another study, however, did not observe significant associations with crowding and leprosy acquisition [15]. The reasons for these discrepancies may reflect the nature of the disease, in particular the lengthy incubation period which may lead to undeestimations of contact risk.

There is a lack of rapid and field-friendly tools for early disease detection. Levels of anti-PGL-1 IgM antibodies showing exposure or infection from *M*.*leprae* could be a candidate tool to support early diagnosis. While evidence is lacking that anti-PGL-I IgM antibodies alone can be used for leprosy infection in HHCs, some studies have observed that anti-PGL-I IgM antibody positive contacts are at increased risk of developing leprosy [5, 16].

An increased level of anti- PGL-I IgM noted in one of our HHC. Despite the opposing views mentioned above, it helped the diagnosis **(Fig 6B).** Our result showed similarity to the study findings of Spencer and his colleagues. Their finding had shown many HHCs with progressively elevated antibody titers. One HHC developed borderline lepromatous leprosy (BL) after two years routine follow-up [6]. But the Bangladesh study opposed the above statement. The study stated its insufficiency when used alone. According to that study, it needs other markers for early detection of leprosy and onset of the disease [4].

In our study PGL-I results decreased after MDT in the majority of our patients and HHCs. This finding is in line with Spencer and Brennan’s study. The study showed decreased seropositivity even after the first MDT administration.This is due to bacilli destruction where the PGL-I synthesis is also reduced. Measuring PGL-I IgM serum level after instilling MDT considered for monitoring. It helps in checking the effectiveness of MDT in leprosy especially in patients with BI >3 [17].

Anti-PGL-I IgM antibodies in adjunct with other biomarkers improved the diagnostic accuracy of PB leprosy, and improved resolution has also been obtained with a combination of both humoral and cellular markers [18, 19]. A point of care multiplex biomarker analytical test using up to 6 biomarkers has also been recently developed which includes anti-PGLI IgM antibodies as a component. It is easy to use under field conditions and has the potential to distinguish both MB and PB leprosy patients from controls [20].

A recent improved test has also been developed by Dijk *et al*., [21]. This point-of-care test uses synthetic phenyl glycolipids as an antigen component and can detect *M*.*leprae* IgM antibodies. The test will be applicable in future field studies in low resource leprosy endemic areas [21].

Several challenges encountered during conducting the ACD included the increased responsibility of the HEWs and knowledge gap on leprosy. Additionally, high turnover and rotation of trained health workers constrained leprosy management. Lack of awareness, stigma and low perception of the community were also challenges. The HEWs program has not been implemented in hospitals and this was an obstacle for the ACD implementation which in our study required individuals experienced in ACD and contact tracing [22].

A limitation of this study was that we were unable to screen all HHCs, 23% were not screened. Political instability in the region was an obstacle and with greater stability we could have been able to improve case detection.

## Conclusion

This study showed that ACD and HHC tracing successfully identified many unsuspected leprosy cases in the Kokosa district during the study period. Through ACD and HHC tracing, prevention of potential disability can be achievable through early diagnosis. Our work is in line with the current leprosy strategy to interrupt transmission “Towards zero leprosy, 2022–2030”.

ACD helps in interrupting transmission of the disease within the family and beyond. Introducing similar efforts in other leprosy hotspot districts are applicable. Thus, ACD and contact tracing are proven strategies, contributing to early detection of leprosy and decreasing transmission. Ethiopia has established a good health extension program (HEP). Through this existing HEP, ACD is applicable with little investment and should be considered by public health authorities as avenue for early and better case finding, contributing to leprosy elimination.

## Supporting information

S1 File (DOCX)
