# Correction: Reaching those at risk: Active case detection of leprosy and contact tracing at Kokosa, a hot spot district in Ethiopia

**DOI:** 10.1371/journal.pone.0342020

**Published:** 2026-01-29

**Authors:** Tsehaynesh Lema, Kidist Bobosha, Christa Kasang, Azeb Tarekegne, Saba Lambert, Addis Mengiste, Sven Britton, Abraham Aseffa, Yimtubezenash Woldeamanuel

Anouk van Hooij is not included in the author byline. Anouk van Hooij should be listed as the seventh author and affiliated with Department of infectious diseases, Leiden University Medical Center, the Netherlands. The contributions of this author are as follows: Developed/designed methodology, and wrote/ reviewed & edited the paper.

Annemieke Geluk is not included in the author byline. Annemieke Geluk should be listed as the eighth author and affiliated with Department of infectious diseases, Leiden University Medical Center, the Netherlands. The contributions of this author are as follows: Developed/designed methodology, and wrote/ reviewed & edited the paper.

In the Biological samples preparation subsection of the Materials and methods, there is an error in the first sentence of the fifth paragraph. The correct sentence is: Positive and negative control serum samples was used to check UCP-LFA performance.

## References

[1] Lema T, Bobosha K, Kasang C, Tarekegne A, Lambert S, Mengiste A, et al. Reaching those at risk: Active case detection of leprosy and contact tracing at Kokosa, a hot spot district in Ethiopia. PLoS One. 2023;18(6):e0264100. doi: 10.1371/journal.pone.0264100 37343000 PMC10284383

