# Supplementary material for: Reaching those at risk: Active case detection of leprosy and contact tracing at Kokosa, a hot spot district in Ethiopia
Source: PLoS One. 2023 Jun 21;18(6):e0264100. doi: 10.1371/journal.pone.0264100 (PMC10284383; doi:10.1371/journal.pone.0264100)
Supplement: S1 File — (DOCX) [file pone.0264100.s001.docx]

| ques_num | study group | age | sex | residence | marital statu | education | occupation | house size |
| --- | --- | --- | --- | --- | --- | --- | --- | --- |
| 1 | 1-Leprosy | 45 | 1-Male | 1-Rural | 1-Married |  | 1-Farmer | 8 |
| 2 |  | 8 | 1-Male | 1-Rural | 0-Single |  | child | 8 |
| 3 |  | 25 | 1-Male | 1-Rural | 1-Married | 3-Secondary (9-1 | 0-Civil servant | 3 |
| 4 |  | 55 | 1-Male | 1-Rural | 1-Married | 0-Not able to rea | 1-Farmer | 9 |
| 5 |  | 30 | 0-Female | 1-Rural | 1-Married | 0-Not able to rea | 4-House wife | 6 |
| 6 |  | 20 | 1-Male | 1-Rural | 1-Married | 2-Primary (1-8) | 3-Student | 7 |
| 7 |  | 28 | 1-Male | 1-Rural | 1-Married | 0-Not able to rea | 1-Farmer | 4 |
| 8 |  | 35 | 0-Female | 1-Rural | 1-Married | 0-Not able to rea | 4-House wife | 6 |
| 9 |  | 30 | 0-Female | 1-Rural | 1-Married | 0-Not able to rea | 4-House wife | 8 |
| 10 |  | 70 | 1-Male | 1-Rural | 1-Married | 0-Not able to rea | 1-Farmer | 7 |
| 11 |  | 23 | 1-Male | 1-Rural | 1-Married | 2-Primary (1-8) | 2-Daily laboure | 8 |
| 12 |  | 25 | 1-Male | 0-Urban | 1-Married | 3-Secondary (9-1 | 6-Merchant | 3 |
| 13 |  | 25 | 1-Male | 1-Rural | 1-Married | 1-Able to read a | 1-Farmer | 4 |
| 14 |  | 19 | 1-Male | 1-Rural | 0-Single | 1-Able to read a | 1-Farmer | 7 |
| 15 |  | 32 | 0-Female | 1-Rural | 1-Married | 0-Not able to rea | 4-House wife | 6 |
| 16 |  | 7 | 1-Male | 1-Rural | 0-Single | 0-Not able to rea | 7-Others | 12 |
| 17 |  | 7 | 1-Male | 1-Rural | 0-Single | 0-Not able to rea | 7-Others | 12 |
| 18 |  | 4 | 0-Female | 1-Rural | 0-Single | 0-Not able to rea | 7-Others | 6 |
| 19 |  | 13 | 1-Male | 1-Rural | 0-Single | 1-Able to read a | 3-Student | 8 |
| 20 |  | 8 | 0-Female | 1-Rural | 0-Single | 0-Not able to rea | 7-Others | 6 |
| 21 |  | 8 | 0-Female | 1-Rural | 0-Single | 0-Not able to rea | 7-Others | 8 |
| 22 |  | 6 | 1-Male | 1-Rural | 0-Single | 0-Not able to rea | 7-Others | 8 |
| 23 |  | 13 | 1-Male | 1-Rural | 0-Single | 2-Primary (1-8) | 3-Student | 5 |
| 24 |  | 10 | 1-Male | 1-Rural | 0-Single | 2-Primary (1-8) | 3-Student | 13 |
| 25 |  | 17 | 0-Female | 1-Rural | 0-Single | 2-Primary (1-8) | 3-Student | 7 |
| 26 |  | 12 | 0-Female | 1-Rural | 0-Single | 2-Primary (1-8) | 3-Student | 5 |
| 27 |  | 55 | 0-Female | 1-Rural | 1-Married | 0-Not able to rea | 1-Farmer | 7 |
| 28 |  | 70 | 1-Male | 1-Rural | 1-Married | 0-Not able to rea | 7-Farmer | 5 |
| 29 |  | 35 | 1-Male | 1-Rural | 1-Married | 0-Not able to rea | 1-Farmer | 6 |
| 30 |  | 55 | 0-Female | 1-Rural | 1-Married | 0-Not able to rea | 4-House wife | 7 |
| 31 |  | 20 | 0-Female | 1-Rural | 1-Married | 2-Primary (1-8) | 4-House wife | 7 |
| 32 |  | 25 | 0-Female | 1-Rural | 1-Married | 0-Not able to read and write\| | | 5 |
| 33 |  | 25 | 1-Male | 1-Rural | 0-Single | 2-Primary (1-8) | 1-Farmer | 3 |
| 34 |  | 51 | 0-Female | 1-Rural | 1-Married | 0-Not able to rea | 4-House wife | 6 |
| 35 |  | 45 | 1-Male | 1-Rural | 1-Married | 0-Not able to rea | 1-Farmer | 13 |
| 36 |  | 40 | 1-Male | 1-Rural | 1-Married | 0-Not able to rea | 1-Farmer | 7 |
| 37 |  | 10 | 1-Male | 1-Rural | 0-Single | 2-Primary (1-8) | 3-Student | 8 |
| 38 |  | 19 | 0-Female | 1-Rural | 0-Single | 2-Primary (1-8) | 3-Student | 9 |
| 39 |  | 25 | 0-Female | 1-Rural | 1-Married | 0-Not able to rea | 4-House wife | 5 |
| 40 |  | 12 | 1-Male | 1-Rural | 0-Single | 2-Primary (1-8) | 3-Student | 9 |
| 41 |  | 21 | 1-Male | 1-Rural | 0-Single | 3-Secondary (9-1 | 3-Student | 2 |
| 42 |  | 25 | 1-Male | 1-Rural | 1-Married | 0-Not able to rea | 1-Farmer | 6 |
| 43 |  | 25 | 1-Male | 1-Rural | 1-Married | 3-Secondary (9-1 | 3-Student | 7 |
| 44 |  | 14 | 0-Female | 1-Rural | 0-Single | 2-Primary (1-8) | 3-Student | 5 |
| 45 |  | 10 | 0-Female | 1-Rural | 0-Single | 0-Not able to rea | 7-Others,Child | 10 |
| 46 |  | 8 | 1-Male | 0-Urban | 0-Single | 2-Primary (1-8) | 3-Student | 6 |

S1 file:Socio-demographic and clinical raw data of patients

| 47 | 28 1-Male | 1-Rural | 0-Single | 0-Not able to rea1-Farmer | 9 |
| --- | --- | --- | --- | --- | --- |
| 48 | 15 0-Female | 1-Rural | 0-Single | 0-Not able to rea1-Farmer | 7 |
| 49 | 50 0-Female | 0-Urban | 3-Widowed | 0-Not able to rea4-House wife | 3 |
| 50 | 26 1-Male | 1-Rural | 0-Single | 2-Primary (1-8) 3-Student | 8 |
| 51 | 20 0-Female | 1-Rural | 1-Married | 3-Secondary (9-14-House wife | 6 |
| 52 | 20 1-Male | 1-Rural | 0-Single | 4-College (10+ o 3-Student | 6 |
| 53 | 30 1-Male | 1-Rural | 1-Married | 0-Not able to rea1-Farmer | 4 |
| 54 | 26 1-Male | 1-Rural | 0-Single | 3-Secondary (9-11-Farmer | 4 |
| 55 | 75 1-Male | 1-Rural | 3-Widowed | 0-Not able to rea1-Farmer | 4 |
| 56 | 30 1-Male | 1-Rural | 1-Married | 0-Not able to rea1-Farmer | 4 |
| 57 | 26 1-Male | 1-Rural | 1-Married | 0-Not able to rea1-Farmer | 8 |
| 58 | 12 1-Male | 1-Rural | 0-Single | 0-Not able to rea7-Others | 6 |
| 59 | 14 1-Male | 1-Rural | 0-Single | 3-Secondary (9-13-Student | 10 |
| 60 | 60 1-Male | 1-Rural | 1-Married | 0-Not able to rea1-Farmer | 11 |
| 61 | 57 0-Female | 1-Rural | 1-Married | 0-Not able to rea4-House wife | 4 |
| 62 | 56 1-Male | 1-Rural | 1-Married | 0-Not able to rea1-Farmer | 5 |
| 63 | 56 0-Female | 1-Rural | 1-Married | 0-Not able to rea4-House wife | 2 |
| 64 | 65 0-Female | 1-Rural | 3-Widowed | 0-Not able to rea1-Farmer | 6 |
| 65 | 70 1-Male | 1-Rural | 3-Widowed | 0-Not able to rea1-Farmer | 10 |
| 66 | 39 0-Female | 1-Rural | 1-Married | 0-Not able to rea1-Farmer | 7 |
| 67 | 70 1-Male | 1-Rural | 1-Married | 0-Not able to rea1-Farmer | 21 |
| 68 | 28 1-Male | 1-Rural | 1-Married | 2-Primary (1-8) 1-Farmer | 5 |
| 69 | 26 1-Male | 1-Rural | 1-Married | 0-Not able to rea1-Farmer | 4 |
| 70 | 18 0-Female | 1-Rural | 0-Single | 2-Primary (1-8) 3-Student | 8 |
| 71 | 17 0-Female | 1-Rural | 0-Single | 0-Not able to read7-others Mental case |  |

**Keys:**

MB + Multibacilliary PB = Paucibaciiliary

G1D= Grade 1 disability G2D= Grade 2 disability PNL= Pure neural leprosy

| health_facility | observed | type_lesions | if_q03othe | feel_pain | feel_num | pro_cloe | go_notsympt |
| --- | --- | --- | --- | --- | --- | --- | --- |
| 2-Health Center | 0-No | 3-Nodules |  | 0-No | 1-Yes | 0-No | 1-Health Center |
| 2-Health Center | 0-No | 2-Macules |  | 0-No | 0- No | 0-No | 1-Health Center |
| 2-Health Center | 1-Yes | 2-Macules |  | 0-No | 1-Yes | 0-No | 1-Health Center |
| 2-Health Center | 1-Yes | 4-Others | PATCHs |  | 1-Yes | 0-No | 1-Health Center |
| 2-Health Center |  | 4-Others | PATCHs | 0-No | 1-Yes | 0-No | 1-Health Center |
| 2-Health Center | 1-Yes | 4-Others | PATCHs | 0-No | 1-Yes |  | 2-Hospital |
| 2-Health Center | 1-Yes | 4-Others | PATCHs | 0-No | 1-Yes | 0-No | 2-Hospital |
| 2-Health Center | 1-Yes | 4-Others | PATCHs | 0-No | 1-Yes | 0-No | 1-Health Center |
| 2-Health Center | 1-Yes | 2-Macules |  | 0-No | 1-Yes |  | 1-Health Center |
| 2-Health Center | 0-No | 2-Macules |  | 0-No | 1-Yes | 0-No | 1-Health Center |
| 1-Hospital | 0-No | 3-Nodules |  | 1-Yes | 1-Yes | 0-No | 2-Hospital |
| 1-Hospital | 0-No | 4-Others | PATCHS | 1-Yes | 1-Yes | 0-No | 2-Hospital |
| 1-Hospital | 1-Yes | 4-Others | PATCHS | 0-No | 1-Yes | 0-No | 2-Hospital |
| 1-Hospital | 0-No | 3-Nodules |  | 1-Yes | 1-Yes | 0-No | 2-Hospital |
| 2-Health Center | 1-Yes | 4-Others | PATCHS | 0-No | 1-Yes | 0-No | 1-Health Center |
| 2-Health Center | 1-Yes | 4-Others | PATCHS | 0-No | 0- No | 0-No | 1-Health Center |
| 2-Health Center | 1-Yes | 4-Others | PATCHS | 0-No | 0- No | 0-No | 1-Health Center |
| 2-Health Center | 1-Yes | 4-Others | PATCHs | 0-No | 0- No | 0-No | 1-Health Center |
| 2-Health Center | 1-Yes | 2-Macules |  | 0-No | 0- No | 0-No | 1-Health Center |
| 2-Health Center | 0-No | 3-Nodules |  | 0-No | 0- No | 0-No | 2-Hospital |
| 2-Health Center | 1-Yes | 4-Others | PATCHS | 0-No | 1-Yes | 0-No | 1-Health Center |
| 2-Health Center | 0-No | 2-Macules |  | 0-No | 0- No | 0-No | 1-Health Center |
| 2-Health Center | 0-No | 4-Others | NEURAL LE | 0-No | 1-Yes | 0-No | 1-Health Center |
| 2-Health Center | 0-No | 2-Macules |  | 0-No | 0- No | 0-No | 1-Health Center |
| 2-Health Center | 1-Yes | 2-Macules |  | 1-Yes | 1-Yes | 0-No | 1-Health Center |
| 2-Health Center | 0-No | 2-Macules |  | 0-No | 0- No | 0-No | 1-Health Center |
| 2-Health Center | 1-Yes | 4-Others | PATCHS | 0-No | 1-Yes | 0-No | 1-Health Center |
| 2- Health center | 1-Yes | 1-Papules |  | 0-No | 1-Yes | 0-No | 1-Health Center |
| 2-Health Center | 1-Yes | 4-Others | PATCHS | 0-No | 1-Yes | 0-No | 1-Health Center |
| 2-Health Center | 0-No | 4-Others | NEURAL LEPROSY | | 1-Yes | 0-No | 1-Health Center |
| 2-Health Center | 0-No | 4-Others | PATCHS | 0-No | 1-Yes | 0-No | 1-Health Center |
| 2-Health Center | 1-Yes | 4-Others | PATCHS | 0-No | 1-Yes | 0-No | 1-Health Center |
| 2-Health Center | 1-Yes | 1-Papules |  | 1-Yes | 1-Yes | 0-No | 1-Health Center |
| 2-Health Center | 1-Yes | 2-Macules |  | 0-No | 1-Yes | 0-No | 1-Health Center |
| 2-Health Center | 1-Yes | 2-Macules |  | 0-No | 1-Yes | 1-Yes | 2-Hospital |
| 2-Health Center | 1-Yes | 2-Macules |  | 0-No | 1-Yes | 0-No | 1-Health Center |
| 2-Health Center | 0-No | 2-Macules |  | 0-No | 1-Yes | 0-No | 1-Health Center |
| 2-Health Center | 1-Yes | 2-Macules |  | 0-No | 0- No | 0-No | 1-Health Center |
| 2-Health Center | 0-No | 4-Others | PATCHS | 0-No | 1-Yes | 1-Yes | 2-Hospital |
| 2-Health Center | 1-Yes | 2-Macules |  | 0-No | 1-Yes | 0-No | 1-Health center |
| 2-Health Center | 1-Yes | 4-Others | PATCHS | 0-No | 1-Yes | 0-No | 2-Hospital |
| 1-Hospital | 0-No | 2-Macules |  | 0-No | 1-Yes | 0-No | 2-Hospital |
| 2-Health Center | 1-Yes | 3-Nodules |  | 0-No | 1-Yes | 0-No | 1-Health Center |
| 2-Health Center | 1-Yes | 4-Others | PATCHS | 0-No | 1-Yes | 0-No | 1-Health Center |
| 2-Health Center | 0-No | 4-Others | PATCHS |  | 1-Yes | 0-No | 1-Health Center |
| 2-Health Center | 1-Yes | 4-Others | PATCHS | 0-No | 1-Yes | 0-No | 1-Health Center |

| 2-Health Center | 0-No | 3-Nodules |  | 1-Yes | 1-Yes | 0-No | 1-Health Center |
| --- | --- | --- | --- | --- | --- | --- | --- |
| 2-Health Center | 1-Yes | 4-Others | PATCHS | 0-No | 0- No | 0-No | 1-Health Center |
| 2-Health Center | 1-Yes | 2-Macules |  | 0-No | 1-Yes | 0-No | 1-Health Center |
| 2-Health Center | 0-No | 4-Others | NEURAL LE | 0-No | 0- No | 1-Yes | 1-Health Center |
| 2-Health Center | 1-Yes | 3-Nodules |  | 0-No | 1-Yes | 0-No | 2-Hospital |
| 1-Hospital | 1-Yes | 4-Others | PATCHS | 0-No | 1-Yes | 0-No | 2-Hospital |
| 2-Health Center | 0-No | 2-Macules |  | 0-No | 0- No | 0-No | 2-Hospital |
| 2-Health Center | 1-Yes | 2-Macules |  | 0-No | 0- No | 0-No | 1-Health Center |
| 2-Health Center | 0-No | 2-Macules |  | 0-No | 1-Yes | 0-No | 1-Health Center |
| 2-Health Center | 0-No | 2-Macules |  | 0-No | 0- No | 0-No | 1-Health Center |
| 1-Hospital | 0-No | 4-Others | NEURAL LE | 0-No | 1-Yes | 0-No | 2-Hospital |
| 1-Hospital | 1-Yes | 2-Macules |  | 0-No | 1-Yes | 0-No | 2-Hospital |
| 2-Health Center | 1-Yes | 4-Others | PATCHS | 0-No | 0- No | 0-No | 2-Hospital |
| 2-Health Center | 0-No | 4-Others | NEURAL LEPROSY | | 1-Yes | 0-No | 1-Health Center |
| 2-Health Center | 0-No | 4-Others | NEURAL LEPROSY | | 1-Yes | 0-No | 1-Health Center |
| 2-Health Center | 0-No | 4-Others | PATCHS |  | 1-Yes | 0-No | 1-Health Center |
| 2-Health Center | 0-No | 4-Others | PATCHS |  | 1-Yes | 1-Yes | 1-Health Center |
| 2-Health Center | 1-Yes | 4-Others | PATCHS | 0-No | 1-Yes | 0-No | 1-Health Center |
| 2-Health Center | 0-No | 4-Others | PATCHS | 0-No | 1-Yes | 0-No | 1-Health Center |
| 2-Health Center | 0-No | 2-Macules |  | 0-No | 0- No | 0-No | 1-Health Center |
| 2-Health Center | 1-Yes | 2-Macules |  | 0-No | 1-Yes | 0-No | 1-Health Center |
| 2-Health Center | 0-No | 4-Others | NEURAL LE | 0-No | 1-Yes | 0-No | 1-Health Center |
| 2-Health Center | 0-No | 2-Macules |  | 0-No | 0- No | 0-No | 2-Hospital |
| 2-Health Center | 1-Yes | 2-Macules |  | 0-No | 0- No | 0-No | 1-Health Center |
| 2-Health Center | 0-No | 2-Macules |  |  |  |  | 1-Health Center |

| leprosy cl | leprosy re | if reaction | closer_con | ifq01yes(h | lepopat_fa | Slit Skin Sm | Disability |
| --- | --- | --- | --- | --- | --- | --- | --- |
| 0-MB | 0-No |  | 0-No |  | 0-No | 5 |  |
| 0-MB | 0-No |  | 0-No |  | 0-No | 0 |  |
| 0-MB | 0-No |  | 0-No |  | 0-No | 4 |  |
| 0-MB | 0-No |  | 0-No |  | 0-No | 0 |  |
| 1-PB | 0-No |  | 0-No |  | 0-No | 0 |  |
| 0-MB | 0-No |  | 0-No |  | 0-No | 0 |  |
| 0-MB | 0-No |  | 0-No |  | 0-No | 4 |  |
| 0-MB | 0-No |  | 0-No |  | 0-No | 0 |  |
| 0-MB | 0-No |  | 0-No |  | 0-No | POS |  |
| 0-MB | 0-No |  | 0-No |  | 0-No | ND |  |
| 0-MB | 1-Yes | 0-Type I | 0-No |  | 0-No | 1 |  |
| 0-MB | 0-No |  | 0-No |  | 0-No | 4 |  |
| 1-PB | 0-No |  | 0-No |  | 0-No | 0 |  |
| 1-PB | 1-Yes | 0-Type I | 0-No |  | 0-No | 0 |  |
| 0-MB | 0-No |  | 0-No |  | 0-No | 0 |  |
| 0-MB | 0-No |  | 1-Yes | 7 | 1-Yes | 0 |  |
| 1-PB | 0-No |  | 1-Yes | 7 | 1-Yes | 0 |  |
| 1-PB | 0-No |  | 0-No |  | 0-No | 0 |  |
| 0-MB | 0-No |  | 1-Yes | 13 | 1-Yes | 1 |  |
| 0-MB | 0-No |  | 0-No |  | 0-No | 4 |  |
| 0-MB | 0-No |  | 1-Yes | 8 | 1-Yes | 0 |  |
| 0-MB | 0-No |  | 1-Yes | 6 | 1-Yes | ND |  |
| 0-MB | 1-Yes | 0-Type I | 0-No |  | 0-No | PNL ND | G2D |
| 0-MB | 0-No |  | 1-Yes | 10 | 1-Yes | ND |  |
| 0-MB | 0-No |  | 0-No |  | 0-No | ND |  |
| 0-MB | 0-No |  | 1-yes | 12 | 1-Yes | 0 |  |
| 0-MB | 0-No |  | 0-No |  | 0-No | 0 | G2D |
| 0-MB | 0-No |  | 0-No |  | 0-No | 0 | G2D |
| 0-MB | 0-No |  | 0-No |  | 0-No | 0 |  |
| 0-MB | 0-No |  | 0-No |  | 0-No | PNL 0 |  |
| 1-PB | 0-No |  | 0-No |  | 0-No | ND | G1D |
| 1-PB | 0-No |  | 0-No |  | 0-No | ND |  |
| 0-MB | 0-No |  | 0-No |  | 0-No | 0 |  |
| 0-MB | 0-No |  | 0-No |  | 0-No | 0 |  |
| 0-MB | 1-Yes | 0-Type I | 0-No |  | 0-No | ND |  |
| 0-MB | 0-No |  | 0-No |  | 0-No | 0 | G2D |
| 0-MB | 0-No |  | 0-No |  | 0-No | 1 |  |
| 1-PB | 0-No |  | 1-Yes | 10 | 1-Yes | 0 |  |
| 0-MB | 1-Yes | 0-Type I | 0-No |  | 0-No | 1 |  |
| 0-MB | 0-No |  | 1-Yes | 12 | 1-Yes | 3 | G2D |
| 0-MB | 0-No |  | 0-No |  | 0-No | 2 |  |
| 0-MB | 0-No |  | 1-Yes | 6 | 1-Yes | 0 | G1D |
| 0-MB | 0-No |  | 1-Yes | 6 | 1-Yes | 0 |  |
| 0-MB | 0-No |  | 0-No |  | 0-No | 0 |  |
| 0-MB | 0-No |  | 0-No |  | 0-No | POS | G2D |
| 0-MB | 0-No |  | 0-No |  | 0-No | 2 |  |

| 0-MB | 0-No |  | 0-No |  | 0-No | 6 | G1D |
| --- | --- | --- | --- | --- | --- | --- | --- |
| 0-MB | 0-No |  | 1-Yes | 15 | 1-Yes | 0 |  |
| 0-MB | 0-No |  | 0-No |  | 0-No | 0 | G2D |
| 0-MB | 1-Yes | 1-Type 2 | 0-No |  | 0-No | PNL ND | G2D |
| 0-MB | 1-Yes | 1-Type 2 | 0-No |  | 0-No | 4 |  |
| 1-PB | 0-No |  | 0-No |  | 0-No | ND |  |
| 0-MB | 0-No |  | 1-Yes | 30 | 1-Yes | 0 | G2D |
| 0-MB | 0-No |  | 0-No |  | 0-No | ND |  |
| 0-MB | 0-No |  | 1-Yes | 30 | 1-Yes | 0 | G2D |
| 0-MB | 0-No |  | 1-Yes | 28 | 1-Yes | 0 |  |
| 0-MB | 0-No |  | 0-No |  | 0-No | PNL ND |  |
| 0-MB | 0-No |  | 1-Yes | 12 | 1-Yes | 0 |  |
| 0-MB | 0-No |  | 1-Yes | 14 | 1-Yes | 1 |  |
| 0-MB | 0-No |  | 1-Yes | 20 | 1-Yes | PNL 0 | G2D |
| 0-MB | 0-No |  | 0-No |  | 0-No | PNL 0 | G2D |
| 1-PB | 0-No |  | 0-No |  | 0-No | ND | G2D |
| 1-PB | 0-No |  | 0-No |  | 0-No | ND | G2D |
| 1-PB | 0-No |  | 0-No |  | 0-No | 0 |  |
| 1-PB | 0-No |  | 0-No |  | 0-No | 0 |  |
| 0-MB | 0-No |  | 1-Yes | 10 | 1-Yes | ND |  |
| 0-MB | 0-No |  | 1-Yes | 30 | 1-Yes | 5 | G2D |
| 0-MB | 0-No |  | 0-No |  | 0-No | PNL ND |  |
| 1-PB | 0-No |  | 0-No |  | 0-No | 0 |  |
| 0-MB | 0-No |  | 0-No |  | 0-No | 0 |  |
| 0-MB | 0-No |  | 1-Yes | 17 | 1-Yes | ND |  |

| **S2 file :PGL-1 raw data of Leprosy patients and their household contacts at baseline and follow up** | | | | | | | |
| --- | --- | --- | --- | --- | --- | --- | --- |
| 2018LP | | 2018MB | 2018PB | HHC_t0 | HHC_t1 | Lep_t0 | Lep_t1 |
| BOK-01-005 | 1.706 | 1.706 |  | 0.082 | 0.068863 | 1.706 | 0.867404 |
| HEB-01-009 | 1.498 | 1.498 |  | 0.022 | 0.036042 | 1.498 | 0.731822 |
| BOK-01-010 | 1.193 | 1.193 |  | 0.096 | 0.158107 | 1.193 | 0.521402 |
| BOK-01-003 | 0.477 | 0.477 |  | 0.43 | 0.104584 | 0.477 | 0.20102 |
| HEB-01-013 | 0.337 | 0.337 |  | 0.205 | 0.138699 | 0.337 | 0.264164 |
| BOK-01-006 | 0.312 | 0.312 |  | 0.15 | 0.061243 | 0.312 | 0.183584 |
| HEB-01-011 | 0.191 | 0.191 |  | 0.18 | 0.109598 | 0.191 | 0.053451 |
| BOK-01-008 | 0.168 | 0.168 |  | 0.025 | 0.060053 | 0.168 | 0.125889 |
| HEB-01-012 | 0.165 | 0.165 |  | 0.087 | 0.07108 | 0.165 | 0.175445 |
| BOR-01-011 | 0.146 | 0.146 |  | 0.594 | 0.113303 | 0.146 | 0.184818 |
| BOK-01-007 | 0.119 | 0.119 |  | 0.15 | 0.086921 | 0.119 | 0.13829 |
| HEB-01-008 | 0.106 | 0.106 |  | 0.25 | 0.092174 | 0.106 | 0.06107 |
| BOR-02-014 | 0.06 |  | 0.06 | 0.072 | 0.132759 | 0.06 | 0.038895 |
| HOG-02-013 | 0.023 |  | 0.023 | 0.1 | 0.092875 | 0.023 | 0.144662 |
|  |  |  |  | 0.312 | 0.19506 |  |  |
| BOK-03-001 |  |  |  | 0.135 | 0.072769 |  |  |
| BOK-03-002 |  |  |  | 0.104 | 0.040076 |  |  |
| BOK-03-003 |  |  |  | 0.121 | 0.073717 |  |  |
| BOK-03-004 |  |  |  | 0.115 | 0.042213 |  |  |
| BOK-03-006 |  |  |  | 0.096 | 0.063311 |  |  |
| BOK-03-007 |  |  |  | 0.22 | 0.132011 |  |  |
| BOK-03-009 |  |  |  | 0.232 | 0.038981 |  |  |
| BOK-03-010 |  |  |  | 0.235 | 0.045903 |  |  |
| BOK-03-011 |  |  |  | 0.064 | 0.088074 |  |  |
| BOK-03-012 |  |  |  | 0.077 | 0.083526 |  |  |
| BOK-03-013 |  |  |  | 0.337 | 0.113533 |  |  |
| BOK-03-014 |  |  |  | 0.064 | 0.05919 |  |  |
| BOR-03-001 |  |  |  | 0.11 | 0.048014 |  |  |
| BOR-03-002 |  |  |  | 0.235 | 0.069317 |  |  |
| BOR-03-003 |  |  |  | 0.052 | 0.022734 |  |  |
| BOR-03-004 |  |  |  | 0.23 | 0.05315 |  |  |
| BOR-03-009 |  |  |  | 0.124 | 0.04907 |  |  |
| BOR-03-010 |  |  |  | 0.438 | 0.199012 |  |  |
| BOR-03-012 |  |  |  | 0.155 | 0.038475 |  |  |
| BOR-03-013 |  |  |  | 0.155 | 0.039157 |  |  |
| BOR-03-014 |  |  |  | 0.039 | 0.0449 |  |  |
| BOR-03-015 |  |  |  | 0.096 | 0.086295 |  |  |
| BOR-03-016 |  |  |  | 0.184 | 0.12142 |  |  |
| BOR-03-018 |  |  |  | 0.061 | 0.038099 |  |  |
| GHF-03-001 |  |  |  | 0.084 | 0.078061 |  |  |
| GHF-03-003 |  |  |  | 0.227 | 0.108476 |  |  |
| GHF-03-006 |  |  |  | 0.065 | 0.012954 |  |  |
| GHF-03-007 |  |  |  | 0.269 | 0.085262 |  |  |
| GHF-03-011 |  |  |  | 0.482 | 0.322079 |  |  |
| GHF-03-013 |  |  |  | 0.135 | 0.028207 |  |  |

| GHF-03-014 | 0.068 | 0.041626 |
| --- | --- | --- |
| GHF-03-015 | 0.061 | 0.06557 |
| GHF-03-016 | 0.772 | 1.072382 |
| HEB-03-001 | 0.344 | 0.216119 |
| HEB-03-002 | 0.138 | 0.031077 |
| HEB-03-004 | 0.099 | 0.104087 |
| HEB-03-005 | 0.2 | 0.042531 |
| HEB-03-006 | 0.168 | 0.034936 |
| HEB-03-007 | 0.152 | 0.028462 |
| HEB-03-008 | 0.14 | 0.077121 |
| HEB-03-009 | 0.111 | 0.068333 |
| HEB-03-010 | 0.195 | 0.054571 |
| HEB-03-012 | 0.189 | 0.237541 |
| HEB-03-013 | 0.396 | 0.075829 |
| HEB-03-014 | 0.035 | 0.028248 |
| HEB-03-016 | 0.12 | 0.074594 |
| HEB-03-017 | 0.105 | 0.056997 |
| HEB-03-018 | 0.086 | 0.104466 |
| HEB-03-019 | 0.194 | 0.186851 |
| HEB-03-023 | 0.479 | 0.161403 |
| HEB-03-025 | 0.43 | 0.093781 |
| HEB-03-027 |  |  |
| HOG-03-001 |  |  |
| HOG-03-002 |  |  |
| HOG-03-003 |  |  |
| HOG-03-004 |  |  |
| HOG-03-007 |  |  |
| HOG-03-011 |  |  |
| HOG-03-012 |  |  |
| HOG-03-014 |  |  |
| KOK-03-001 |  |  |
| KOK-03-002 |  |  |
| KOK-03-004 |  |  |
| KOK-03-005 |  |  |
| KOK-03-006 |  |  |
| KOK-03-013 |  |  |
